# The impact of anti-tumor approaches on the outcomes of cancer patients with COVID-19: a meta-analysis based on 52 cohorts incorporating 9231 participants

**DOI:** 10.1186/s12885-022-09320-x

**Published:** 2022-03-04

**Authors:** Qing Wu, Shuimei Luo, Xianhe Xie

**Affiliations:** 1 Department of Oncology, Molecular Oncology Research Institute, The First Affiliated Hospital of Fujian Medical University, No. 20, Chazhong Road, Fuzhou, 350005 Fujian China; 2 Fujian Key Laboratory of Precision Medicine for Cancer, The First Affiliated Hospital of Fujian Medical University, Fuzhou, 350005 Fujian China

**Keywords:** Anti-tumor therapy, cancer, COVID-19, Chemotherapy, Solid tumor

## Abstract

**Background:**

This study was designed to investigate the impact of anti-tumor approaches (including chemotherapy, targeted therapy, endocrine therapy, immunotherapy, surgery and radiotherapy) on the outcomes of cancer patients with COVID-19.

**Methods:**

Electronic databases were searched to identify relevant trials. The primary endpoints were severe disease and death of cancer patients treated with anti-tumor therapy before COVID-19 diagnosis. In addition, stratified analyses were implemented towards various types of anti-tumor therapy and other prognostic factors. Furthermore, odds ratios (ORs) were hereby adopted to measure the outcomes with the corresponding 95% confidence intervals (CIs).

**Results:**

As indicated in the study consisting of 9231 individuals from 52 cohorts in total, anti-tumor therapy before COVID-19 diagnosis could elevate the risk of death in cancer patients (OR: 1.21, 95%CI: 1.07–1.36, *P* = 0.0026) and the incidence of severe COVID-19 (OR: 1.19, 95%CI: 1.01–1.40, *P* = 0.0412). Among various anti-tumor approaches, chemotherapy distinguished to increase the incidence of death (OR = 1.22, 95%CI: 1.08–1.38, *P* = 0.0013) and severe COVID-19 (OR = 1.10, 95%CI: 1.02–1.18, *P* = 0.0165) as to cancer patients with COVID-19. Moreover, for cancer patients with COVID-19, surgery and targeted therapy could add to the risk of death (OR = 1.27, 95%CI: 1.00–1.61, *P* = 0.0472), and the incidence of severe COVID-19 (OR = 1.14, 95%CI: 1.01–1.30, *P* = 0.0357) respectively. In the subgroup analysis, the incidence of death (OR = 1.17, 95%CI: 1.03–1.34, *P* = 0.0158) raised in case of chemotherapy adopted for solid tumor with COVID-19. Besides, age, gender, hypertension, COPD, smoking and lung cancer all served as potential prognostic factors for both death and severe disease of cancer patients with COVID-19.

**Conclusions:**

Anti-tumor therapy, especially chemotherapy, augmented the risk of severe disease and death for cancer patients with COVID-19, so did surgery for the risk of death and targeted therapy for the incidence of severe COVID-19.

**Supplementary Information:**

The online version contains supplementary material available at 10.1186/s12885-022-09320-x.

## Background

As is known to all, the sudden outbreak and global overrun of coronavirus disease 2019 (COVID-19), caused by severe acute respiratory syndrome-related coronavirus 2 (SARS-CoV-2) [1], have generated heavy burdens and great challenges to global public health since December 2019 [2]. Up to date, people all over the world have been fighting against the fatal disease, as reported in over 200 million infected individuals.

Cancer patients are generally in severe immunosuppressive status deriving from cancer itself and the anti-tumor regimens. Furthermore, they have to visit the hospital regularly for monitoring or anti-tumor treatment (such as chemotherapy, immunotherapy, endocrine therapy, targeted therapy, surgery and radiotherapy) leading to increasing exposure to virus.

A growing number of studies revealed that, during the pandemic, cancer patients with COVID-19 generally suffered from worse outcomes compared to patients with COVID-19 alone [3–7]. In addition, some investigations targeted at exploring whether anti-tumor therapy was an additional risk factor for adverse outcomes of COVID-19 and whether it was necessary to change therapeutic modalities to mitigate the risk [8–10].

As far as we know, accumulating prospective and retrospective studies were conducted to evaluate clinical characteristics of cancer patients with COVID-19, as well as the impact of anti-tumor therapy on clinical outcomes of COVID-19 [11–13]. Nevertheless, research findings remained to be a bit conflicting and inconclusive as for the impact of anti-tumor therapeutic approaches on the severity of COVID-19 [14–18]. Consequently, a comprehensive survey based on a larger scale (52 cohorts incorporating 9231 individuals) and diverse dimensions was hereby carried out to clarify the correlation between anti-tumor therapy and COVID-19 prognosis.

## Methods

### Data sources and literature searches

A systematic electronic literature retrieval was in place for study screening, searching for abstracts of relevant studies in the published literature. PubMed, Cochrane Library and EMBASE were all searched with data updated as of 27th March 2021. Basic search terms entered were as follows: “COVID-19”, “SARS-CoV2”, “SARS-CoV-2”, “2019-nCoV”, “novel coronavirus”, “cancer”, “neoplasm”, “malignancy”, “carcinoma” and “tumor” (the full search strategy as shown in Additional file 1: Appendix 1). In addition, full-text papers were scrutinized as for abstracts without substantial information, and the references of relevant articles were reviewed for additional studies. Data retrieval was completed in English, with reviews, editorials comments and case reports all excluded.

### Selection of studies and definition

Initially, two investigators performed a screening of titles and abstracts respectively, then examined the full-text of articles to acquire eligible studies. Regarding the duplicate studies based on the same patients, only the latest or most comprehensive data were recruited as a whole.

Definition:

Anti-tumor therapy: patients receiving chemotherapy (cytotoxic chemotherapy), immunotherapy (immune checkpoint inhibitor), targeted therapy (molecular targeted therapy), surgery, radiotherapy, endocrine therapy (hormonal drugs) within the last 6 months before COVID-19 diagnosis.

Age: defined as “old” or “young” depending on each cut-off used to calculate the odds ratios (ORs) of age in the included studies.

Eastern Cooperative Oncology Group Performance Scale (ECOG PS): defined as “high” or “low” with a cut-off of 2.

Comorbidities: defined as “yes” or “no” to identify cancer patients with or without hypertension, diabetes, chronic obstructive pulmonary disease (COPD), cardiovascular disease, obesity status and smoking in the corresponding studies.

Blood parameters: defined as “high” or “normal” on the basis of each cut-off applied to calculate the ORs of white blood cell count, C-reactive protein (CRP), lymphocyte count, D-dimer, neutrophil to lymphocyte ratio (NLR), and creatine kinase in each included study.

Severe COVID-19: depending on respective definitions in the included studies, including infections requiring intensive care unit (ICU) admission, mechanical ventilation or even resulting in death.

### Inclusion criteria

1) Prospective or retrospective studies to evaluate the impact of anti-tumor therapy on cancer patients with COVID-19; 2) patients pathologically confirmed as cancer; 3) patients diagnosed as COVID-19; 4) studies with data available for ORs and corresponding 95% confidence intervals (CIs) of severe COVID-19 and death rates in groups receiving anti-tumor treatments or not.

### Data extraction

In this study, data extraction was implemented strictly according to the PRISMA guidelines (as shown in Additional file 2: Appendix 2). Meanwhile, all eligible studies involved the information as follows: the publication year and region, first author’s name, study type, number of patients, anti-tumor therapy, severe COVID-19 and/or death cases.

### Quality assessment

The quality of included studies was assessed independently by two reviewers using the Newcastle-Ottawa Scale (NOS) for case-control and cohort studies, encompassing three dimensions of selection, comparability and exposure, with a full score of 9 points.

### Statistical methods

The primary endpoints were composed of death and/or severe COVID-19 of cancer patients treated with anti-tumor therapy before COVID-19 diagnosis. Moreover, the correlation between anti-tumor therapy and the outcomes was determined by ORs with the corresponding 95%CIs. Subgroup analyses were further accomplished based on the type of anti-tumor therapy, type of cancer (solid cancer or haematological malignancy) and other prognostic factors. In addition, funnel plots and Egger’s test were applied to evaluate publication bias, and statistical analysis was realized via R 4.0 statistical software. Heterogeneity was assessed by means of I-square tests and chi-square, with remarkable heterogeneity in case of *P* < 0.1 or *I*^2^ > 40%. Furthermore, a random effect model was adopted to analyze the pooled data when heterogeneity existed; otherwise, a fixed effect model was employed accordingly.

## Results

### Selection of study

**Fig. 1:**
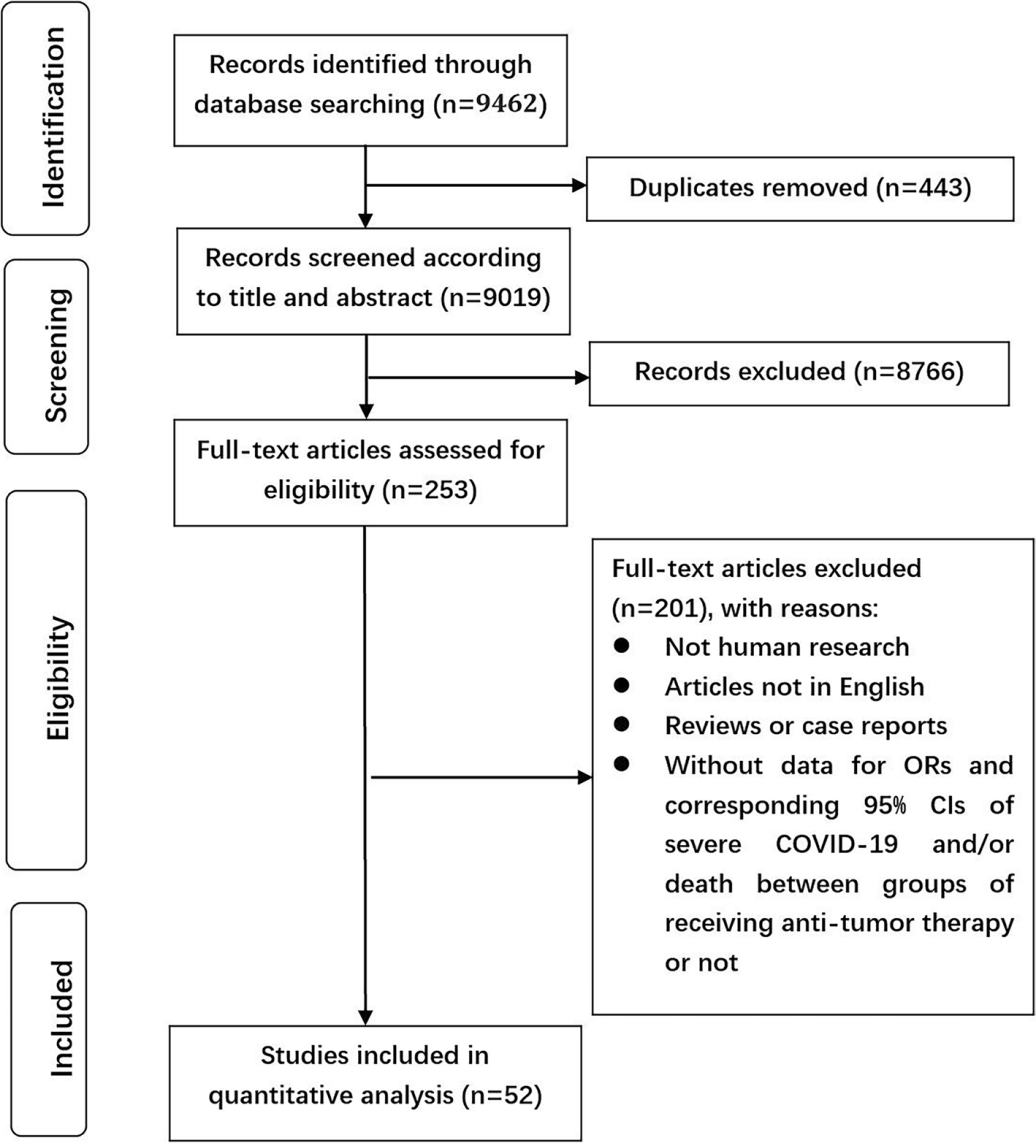
Flowchart on selection including trials in the meta-analysis

Initially, 9462 relevant articles were scrutinized intensively, of which 443 were filtered for duplication, and 8766 were excluded for digression after screening the titles and abstracts. After that, the full text of remaining 253 articles was thoroughly reviewed, among which 201 were excluded as they were reviews or case reports, not human research, not in English, without data for ORs and corresponding 95%CIs of severe COVID-19 and/or death in groups receiving anti-tumor therapy or not. Finally, a total of 52 cohorts [4, 6, 7, 11, 12, 14–60] incorporating 9231 participants were recruited in this study. See Fig. 1 for detailed procedures.

### Study traits

As of 27th March 2021, altogether 9231 individuals in 52 cohorts were included with a sample size ranging from 12 to 1289, of which 45 were retrospective, 4 prospective and 3 retro-prospective. Meanwhile, ORs for severe COVID-19 and/or death were utilized to assess the impact of anti-tumor approaches on cancer patients with COVID-19. Among the foregoing studies, 41 cohorts witnessed death and 23 confronted with severe COVID-19. See Table 1 for principal characteristics.

**Table 1:** The principal characteristics and further details of eligible articles

**Author**	**Year**		**Study design**	**Region**		**Number** **of** **patient**	**Male**	**Median** **age** **(IQR)** **(years)**	**Diagnosis method for COVID-19**	**Cancer** **type**		**Comparison group**
Kuderer NM [6]	2020		Retro-prospective	multi-national		928	468	66 (57–76)	RT-PCR	non-specific		cancer patients with no treatment
Lee LYW [19]	2020		Prospective	UK		800	449	69 (59–76)	RT-PCR	non-specific		cancer patients with no treatment
Zhang L [14]	2020		Retrospective	China		28	17	65 (56–70)	RT-PCR	solid tumor		cancer patients with no treatment
Stroppa EM [20]	2020		Retrospective	Italy		25	20	71 (mean) (50–84)	RT-PCR	non-specific		cancer patients with no treatment
Yang K [7]	2020		Retrospective	China		205	96	63 (56–70)	RT-PCR	non-specific		cancer patients with no treatment
Zhang H [21]	2020		Retrospective	China		107	60	66 (36–98)	RT-PCR and/or radiology	non-specific		cancer patients with no treatment
Robilotti EV [22]	2020		Retrospective	USA		423	212	NA	RT-PCR	non-specific		cancer patients with no treatment
Yarza R [23]	2020		Prospective	Spain		63	34	NA	RT-PCR and/or radiology	solid tumor		cancer patients treated other options
Li Q [24]	2020		Retrospective	China		59	31	63 (54–70)	RT-PCR	non-specific		cancer patients with no treatment
Jee J [25]	2020		Retrospective	USA		309	159	NA	RT-PCR	non-specific		cancer patients with no treatment
Sanchez-Pina JM [26]	2020		Retrospective	Spain		39	23	64 (mean)	RT-PCR	hematological malignancies		cancer patients with no treatment
Pinato DJ [15]	2020		Retrospective	multi-national		890	503	68 (mean)	RT-PCR	non-specific		cancer patients with no treatment
Assaad S [27]	2020		Retrospective	France		55	26	64 (mean)	RT-PCR	non-specific		cancer patients with no treatment
Garassino MC [28]	2020		Retrospective	multi-national		200	141	68 (61–75)	RT-PCR	Thoracic Cancer		cancer patients with no treatment
Liang WH [29]	2020		Retrospective	China		18	12	60 (47–87)	RT-PCR	non-specific		cancer patients with no treatment
Ma J [30]	2020		Retrospective	China		37	20	62 (IQR: 59–70)	RT-PCR and/or antibody test	solid tumor		cancer patients with no treatment
Mehta V [11]	2020		Retrospective	USA		218	127	69 (10–92)	RT-PCR	non-specific		cancer patients with no treatment
Yu J [31]	2020		Retrospective	China		12	10	66 (48–78)	RT-PCR and/or CT	solid tumor		cancer patients with no active antitumor treatment
Tian J [4]	2020		Retrospective	China		232	119	64 (58–69)	RT-PCR	non-specific		cancer patients with surgery
Fox TA [32]	2020		Retrospective	UK		55	38	63 (23–88)	RT-PCR, CT, and clinical features	hematological malignancies		cancer patients with no treatment
Booth S [33]	2020		Prospective	UK		66	41	73 (IQR: 63–81)	RT-PCR, radiological, and clinical features	hematological malignancies		cancer patients with no treatment
Cattaneo C [34]	2020		Retrospective	Italy		102	66	68 (mean)	RT-PCR	hematological malignancies		cancer patients with no treatment
Lara OD [35]	2020		Retrospective	USA		121	NA	64 (IQR: 51–73)	RT-PCR and CT	gynecologic cancer		cancer patients with no treatment
Liu C [36]	2020		Retrospective	China		216	113	63 (IQR: 57–70)	RT-PCR	solid tumor		cancer patients with no treatment
Luo J [37]	2020		Retrospective	USA		102	49	68 (IQR: 61–75)	RT-PCR	lung cancer		cancer patients with no treatment
Mato AR [38]	2020		Retrospective	multi-national		198	125	63 (35–92)	RT-PCR	chronic lymphocytic leukemia		cancer patients with no treatment
Rogado J [39]	2020		Retrospective	Spain		45	30	71 (34–90)	RT-PCR	non-specific		cancer patients with no treatment
Russell B [40]	2020		Retro-prospective	UK		156	90	65 (mean)	RT-PCR	solid tumor		cancer patients with no treatment
Scarfò L [41]	2020		Retrospective	multi-national		190	126	72 (48–94)	RT-PCR	chronic lymphocytic leukemia		cancer patients with no treatment
Vuagnat P [42]	2020		Retrospective	France		58	NA	58 (IQR:48–68)	RT-PCR and/or CT	breast cancer		cancer patients with no treatment
Wang BO [43]	2020		Retrospective	USA		58	30	67	RT-PCR	multiple myeloma		cancer patients with no treatment
Wang J [44]	2020		Retrospective	China		283	141	63 (IQR: 55–70)	RT-PCR	non-specific		cancer patients with no treatment
Gonzalez-cao M [45]	2020		Retrospective	Spain		50	27	69 (6–94)	clinical or RT-PCR	melanoma		cancer patients with no treatment
De Melo AC [46]	2020		Retrospective	Brazil		181	71	55 (2–88)	RT-PCR	non-specific		cancer patients with no active antitumor treatment
Albiges L [47]	2020		Retrospective	France		178	76	61 (52–71)	RT-PCR and/or CT	non-specific		cancer patients with no treatment
Martínez-López J [48]	2020		Retrospective	Spain		167	95	71 (IQR: 62–78)	RT-PCR	multiple myeloma (MM)		cancer patients with no treatment
Martín-Moro F [49]	2020		Retrospective	Spain		34	19	72.5 (35–94)	RT-PCR and/or CT	hematological malignancies		cancer patients with no treatment
Lattenist R [50]	2021		Retrospective	Belgium		13	10	70 (IQR: 59–79)	RT-PCR and/or CT	hematological malignancies		cancer patients with no treatment
Nakamura S [51]	2020		Retrospective	Japan		32	22	74.5 (24–90)	RT-PCR	non-specific		cancer patients with no treatment
Rogiers A [52]	2021		Retrospective	multi-national		110	72	63 (27–86)	RT-PCR	non-specific		cancer patients with no treatment
Glenthøj A [16]	2021		Prospective	Denmark		66	40	66.7 (25–91)		hematological malignancies		cancer patients with no treatment
Song C [17]	2020		Retrospective	China		223	116	63 (56–71)	RT-PCR	non-specific		cancer patients with discontinous treatment
Lunski MJ [18]	2020		Retrospective	USA		312	142	NA	RT-PCR	non-specific		cancer patients with no treatment
Nie L [53]	2020		Retrospective	China		45	31	66 (58–74)	RT-PCR	lung cancer		cancer patients with no treatment
Larfors G [54]	2020		Retrospective	Sweden		NA	NA	NA	RT-PCR	non-specific		cancer patients with no treatment
H€ollein A [55]	2020		Retrospective	Germany		17	8	73 (27–82)	RT-PCR	non-specific		cancer patients with no treatment
Garnett C [56]	2020		Retrospective	UK		32	21	72.5 (46–96)	RT-PCR	hematological malignancies		cancer patients with no treatment
Hanna GJ [57]	2020		Retrospective	USA		32	20	70 (38–91)	RT-PCR	head and neck cancer		cancer patients with no treatment
Lie’vre A [58]	2020		Retro-prospective	France		1289	795	67 (19–100)	RT-PCR	solid tumor		cancer patients with no treatment
Smith M [59]	2020		Retrospective	USA		86	NA	69 (mean)	RT-PCR	solid tumor		cancer patients with no treatment
Wu YG [60]	2020		Retrospective	China		14	9	37 (14–68)	RT-PCR	hematological malignancies		cancer patients with no treatment
Yang F [12]	2020		Retrospective	China		52	28	63 (34–98)	RT-PCR	solid tumor		cancer patients with no treatment
**Author**	**Number of the control**	**Anti-tumor therapy**	**Chemotherapy**	**Immunotherapy**	**Targeted therapy**	**Endocrine therapy**	**Surgery**	**Radiotherapy**	**Outcome**	**Required mechanical ventilation**	**Severe COVID-19**	**Death**
Kuderer NM [6]	553	366	160	38	75	85	32	12	death	116	242	121
Lee LYW [19]	272	528	281	44	72	64	29	76	death	NA	360	226
Zhang L [14]	22	6	3	1	2	NA	NA	1	sever COVID-19	10	15	8
Stroppa EM [20]	13	12	8	4	NA	NA	NA	NA	death	NA	NA	9
Yang K [7]	128	54	31	4	12	NA	4	9	death	32	52	40
Zhang H [21]	70	37	NA	6	NA	NA	NA	NA	death	NA	56	23
Robilotti EV [22]	NA	NA	191	31	NA	NA	31	NA	sever COVID-19	40	85	51
Yarza R [23]	NA	NA	36	8	7	10	NA	NA	sever COVID-19; death	NA	24	16
Li Q [24]	43	16	12	NA	6	NA	1	1	death	27	35	16
Jee J [25]	43	170	102	18	49	NA	NA	NA	sever COVID-19	NA	120	31
Sanchez-Pina JM [26]	15	24	4	NA	5	NA	NA	NA	death	NA	18	NA
Pinato DJ [15]	403	479	206	56	93	92	NA	33	sever COVID-19; death	97	565	299
Assaad S [27]	26	29	16	3	14	NA	NA	NA	death	NA	NA	30
Garassino MC [28]	58	142	48	34	28	NA	NA	NA	death	9	NA	66
Liang WH [29]	14	4	NA	NA	NA	NA	NA	NA	sever COVID-19	NA	9	NA
Ma J [30]	24	13	NA	NA	NA	NA	NA	NA	sever COVID-19	NA	20	5
Mehta V [11]	NA	NA	42	5	NA	NA	NA	49	death	45	NA	61
Yu J [31]	5	7	5	2	1	NA	1	4	sever COVID-19; death	NA	3	3
Tian J [4]	NA	NA	NA	NA	NA	NA	119	NA	sever COVID-19	NA	148	NA
Fox TA [32]	NA	NA	29	25	NA	NA	NA	NA	sever COVID-19; death	NA	25	19
Booth S [33]	29	37	NA	NA	NA	NA	NA	NA	death	NA	NA	34
Cattaneo C [34]	43	59	20	28	NA	NA	NA	NA	death	NA	NA	40
Lara OD [35]	NA	NA	NA	NA	NA	NA	NA	NA	death	NA	20	NA
Liu C [36]	138	78	NA	NA	NA	NA	NA	NA	death	NA	NA	37
Luo J [37]	48	54	NA	NA	NA	NA	NA	NA	sever COVID-19; death	18	NA	25
Mato AR [38]	79	119	51	NA	NA	NA	NA	NA	death	53	NA	66
Rogado J [39]	15	30	19	1	2	NA	NA	NA	death	NA	29	19
Russell B [40]	18	81	45	7	5	NA	NA	NA	sever COVID-19; death	NA	28	34
Scarfò L [41]	73	116	NA	NA	NA	NA	NA	NA	sever COVID-19; death	NA	151	56
Vuagnat P [42]	NA	NA	29	NA	19	19	3	36	sever COVID-19	NA	NA	4
Wang BO [43]	11	47	NA	NA	28	NA	NA	NA	death	NA	NA	14
Wang J [44]	188	95	46	NA	12	NA	23	NA	sever COVID-19; death	NA	NA	50
Gonzalez-cao M [45]	12	38	NA	22	16	NA	NA	NA	sever COVID-19; death	NA	34	13
De Melo AC [46]	16	165	63	NA	NA	20	12	10	death	34	NA	60
Albiges L [47]	61	117	66	19	30	16	NA	NA	sever COVID-19; death	NA	47	31
Martínez-López J [48]	NA	NA	83	NA	NA	NA	NA	NA	death	15	141	56
Martín-Moro F [49]	NA	19	NA	NA	NA	NA	NA	NA	death	4	17	11
Lattenist R [50]	6	7	3	NA	NA	NA	NA	NA	death	NA	NA	6
Nakamura S [51]	19	13	10	3	NA	4	13	NA	death	3	NA	11
Rogiers A [52]	NA	NA	25	NA	NA	NA	NA	NA	sever COVID-19; death	NA	35	18
Glenthøj A [16]	10	9	NA	NA	NA	NA	NA	NA	sever COVID-19	NA	33	NA
Song C [17]	19	204	NA	NA	NA	NA	NA	NA	sever COVID-19	NA	159	NA
Lunski MJ [18]	256	56	12	4	9	44	5	2	death	NA	NA	66
Nie L [53]	34	11	4	4	NA	NA	3	NA	death	3	23	11
Larfors G [54]	NA	NA	NA	NA	NA	NA	NA	NA	sever COVID-19; death	NA	NA	NA
H€ollein A [55]	2	15	14	1	2	NA	NA	1	death	3	NA	6
Garnett C [56]	10	22	NA	NA	NA	NA	NA	NA	death	NA	NA	18
Hanna GJ [57]	26	6	3	1	0	NA	4	1	death	NA	NA	NA
Lie’vre A [58]	NA	NA	577	110	181	57	56	133	death	49	NA	370
Smith M [59]	47	39	NA	NA	NA	NA	NA	NA	sever COVID-19	NA	29	NA
Wu YG [60]	NA	NA	7	NA	NA	NA	NA	NA	death	NA	NA	6
Yang F [12]	NA	NA	6	1	NA	NA	2	NA	sever COVID-19	NA	19	11

*Abbreviations*: *ICIs* Immune checkpoint inhibitors, *RT-PCR* Reverse transcription-polymerase chain reaction, *NA* Not available, *ICU* Intensive Care Unit

### Assessment of study quality and publication bias

Refer to Additional file 3: Appendix 3 for quality assessment of 52 recruited studies. Furthermore, no publication bias was defined via Egger’s tests in the pooled analyses for various anti-tumor approaches (see Additional file 4: Appendix 4) and supernumerary prognostic factors (see Additional file 5: Appendix 5).

### Data analysis

**Fig. 2:**
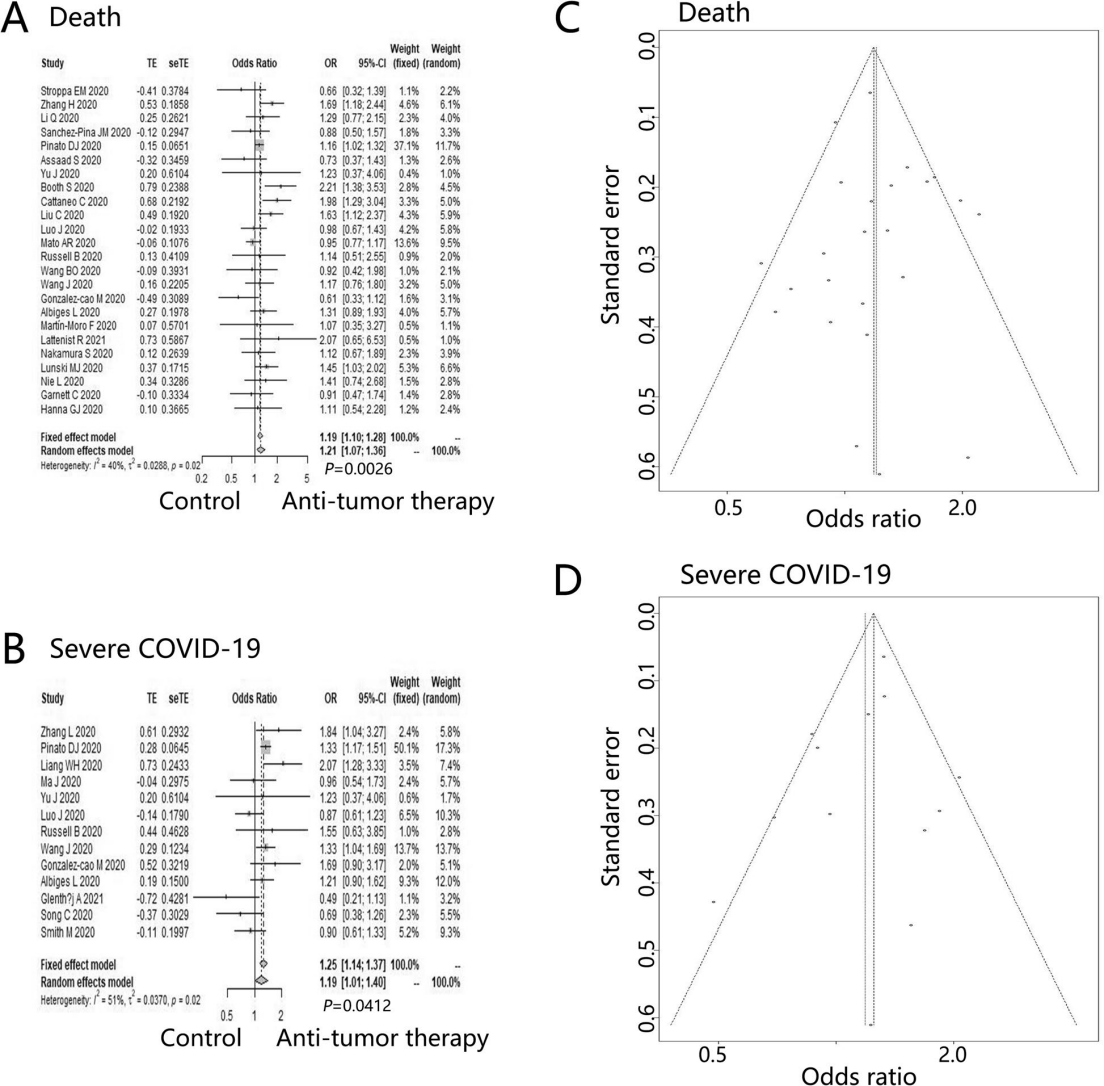
The impact of anti-tumor therapy on clinical outcomes of cancer patients with COVID-19. Forest plots of (**A**) death, **B** severe COVID-19 between groups divided by receiving anti-tumor therapy or not before COVID-19 diagnosis; Funnel plots of (**C**) death, **D** severe COVID-19 between groups divided by receiving anti-tumor therapy or not before COVID-19 diagnosis

In this study, regarding cancer patients treated with anti-tumor therapy before COVID-19 diagnosis, the pooled OR was 1.21 (95%CI: 1.07–1.36, *P* = 0.0026) (Fig. 2A) for death without publication bias (Fig. 2C, Egger’s test: *P* = 0.5516), and 1.19 (95%CI: 1.01–1.40, *P* = 0.0412) (Fig. 2B) for severe COVID-19 without publication bias (Fig. 2D, Egger’s test: *P* = 0.3930).

### The impact of anti-tumor therapy on death and severe disease of cancer patients with COVID-19

**Fig. 3:**
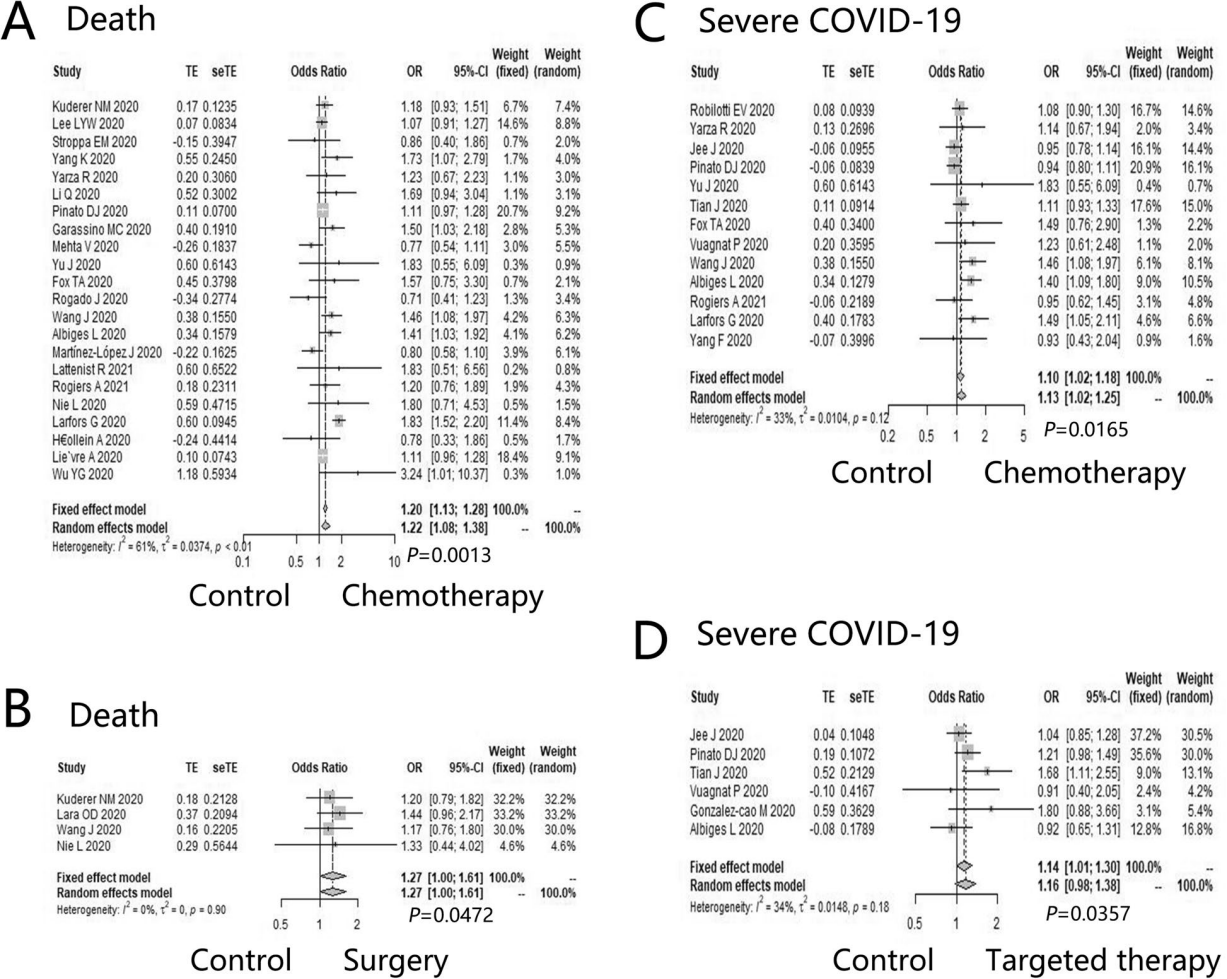
The impact of various anti-tumor approaches on clinical outcomes of cancer patients with COVID-19. The impact of (**A**) chemotherapy and (**B**) surgery on death of cancer patients with COVID-19; The impact of (**C**) chemotherapy and (**D**) targeted therapy on severe disease of cancer patients with COVID-19

As for cancer patients with COVID-19, compared with patients without anti-tumor approaches, the incidence of death appeared to be higher in patients treated with chemotherapy (OR = 1.22, 95%CI: 1.08–1.38, *P* = 0.0013) (Fig. 3A) and surgery (OR = 1.27, 95%CI: 1.00–1.61, *P* = 0.0472) (Fig. 3B), but not in patients receiving radiotherapy (OR = 0.90, 95%CI: 0.75–1.09, *P* = 0.2817), targeted therapy (OR = 0.97, 95%CI: 0.76–1.23, *P* = 0.7914), endocrine therapy (OR = 0.95, 95%CI: 0.80–1.12, *P* = 0.5097), and immunotherapy (OR = 1.05, 95%CI: 0.90–1.22, *P* = 0.5412) (Additional file 6: Appendix 6).

Compared with cancer patients without anti-tumor approaches, the incidence of severe COVID-19 was higher in patients receiving chemotherapy (OR = 1.10, 95%CI: 1.02–1.18, *P* = 0.0165) (Fig. 3C) and targeted therapy (OR = 1.14, 95%CI: 1.01–1.30, *P* = 0.0357) (Fig. 3D), but not in patients treated with surgery (OR = 1.15, 95%CI: 0.89–1.47, *P* = 0.2888) and immunotherapy (OR = 1.18, 95%CI: 0.97–1.45, *P* = 0.1034) (Additional file 6: Appendix 6).

#### Subgroup analysis

Patients were further divided into groups of solid tumor and haematological malignancy depending on the type of cancer, as listed in Table 2. Compared with patients without anti-tumor approaches, solid tumor patients with COVID-19 witnessed higher incidence of death after receiving chemotherapy (OR = 1.17, 95%CI: 1.03–1.34, *P* = 0.0158), but not the case in haematological malignancy patients with COVID-19 (OR = 1.41, 95%CI: 0.74–2.68, *P* = 0.2964).

**Table 2:** Subgroup analysis of the impact of anti-tumor therapy on death and severe disease of cancer patients with COVID-19

Anti-tumor therapy	Solid tumour				Haematological malignancy			
	death		severe COVID-19		death		severe COVID-19	
	OR (95%CI)	***P***	OR (95%CI)	***P***	OR (95%CI)	***P***	OR (95%CI)	***P***
Chemotherapy	1.17 (1.03–1.34)	0.0158	1.16 (0.81–1.66)	0.4072	1.41 (0.74–2.68)	0.2964	NA	NA
Radiotherapy	NA	NA	NA	NA	NA	NA	NA	NA
Targeted therapy	NA	NA	NA	NA	NA	NA	NA	NA
Surgery	NA	NA	NA	NA	NA	NA	NA	NA
Endocrine therapy	NA	NA	NA	NA	NA	NA	NA	NA
Immunotherapy	0.91 (0.47–1.76)	0.7705	NA	NA	NA	NA	NA	NA
Antitumor therapy	1.15 (0.94–1.42)	0.1815	1.08 (0.88–1.32)	0.4643	1.26 (0.91–1.75)	0.1597	NA	NA

*Abbreviations NA* Not available, *OR* Odds ratio, *CI* Confidence interval

### Supernumerary prognostic factors for death and severe disease of cancer patients with COVID-19

**Fig. 4:**
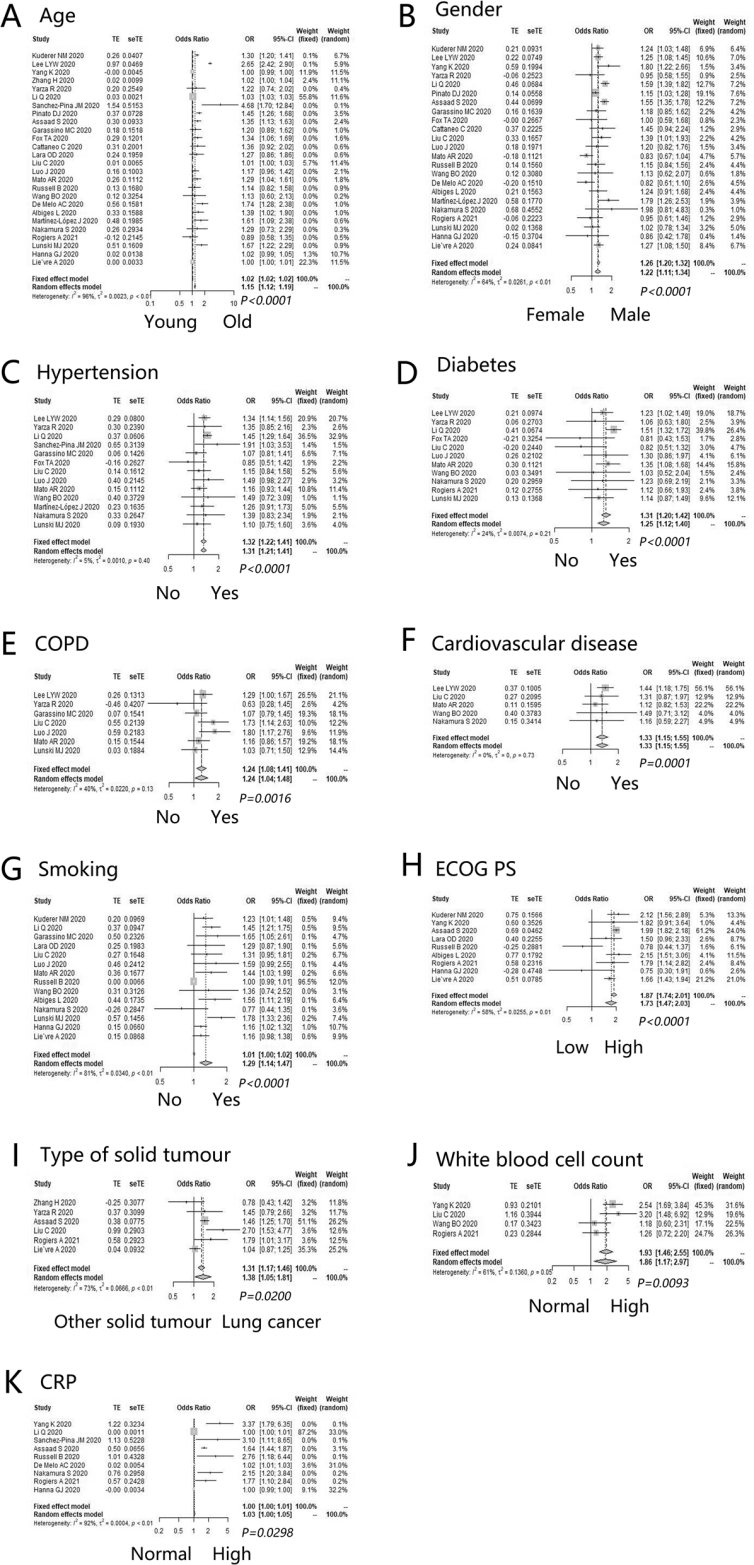
The supernumerary prognostic factors for death of cancer patients with COVID-19. **A** Age (old vs. young); **B** Gender (male vs. female); **C** Hypertension (yes vs. no); **D** Diabetes (yes vs. no); **E** Chronic obstructive pulmonary disease (COPD) (yes vs. no); **F** Cardiovascular disease (yes vs. no); **G** Smoking (yes vs. no); **H** Eastern Cooperative Oncology Group Performance Scale (ECOG PS) (high vs. low); **I** Type of solid tumor (lung cancer vs. other solid tumor); **J** White blood cell count (high vs. normal); **K** C-reactive protein (high vs. normal)

The potential prognostic factors for the death of cancer patients with COVID-19 were as follows: age (OR = 1.15, 95%CI: 1.12–1.19, *P* < 0.0001) (Fig. 4A), gender (OR = 1.22, 95%CI: 1.11–1.34, *P* < 0.0001) (Fig. 4B), hypertension (OR = 1.32, 95%CI: 1.22–1.41, *P* < 0.0001) (Fig. 4C), diabetes (OR = 1.31, 95%CI: 1.20–1.42, *P* < 0.0001) (Fig. 4D), COPD (OR = 1.24, 95%CI: 1.08–1.41, *P* = 0.0016) (Fig. 4E), cardiovascular disease (OR = 1.33, 95%CI: 1.15–1.55, *P* = 0.0001) (Fig. 4F), smoking (OR = 1.29, 95%CI: 1.14–1.47, *P* < 0.0001) (Fig. 4G), ECOG PS (OR = 1.73, 95%CI: 1.47–2.03, *P* < 0.0001) (Fig. 4H), lung cancer (OR = 1.38, 95%CI: 1.05–1.81, *P* = 0.0200) (Fig. 4I), white blood cell count (OR = 1.86, 95%CI: 1.17–2.97, *P* = 0.0093) (Fig. 4J), and CRP (OR = 1.03, 95%CI: 1.00–1.05, *P* = 0.0298) (Fig. 4K). Nevertheless, obesity status (OR = 1.02, 95%CI: 0.91–1.15, *P* = 0.6827), lymphocyte count (OR = 1.24, 95%CI: 0.57–2.68, *P* = 0.5868), D-dimer (OR = 1.01, 95%CI: 0.98–1.05, *P* = 0.3981) and NLR (OR = 1.30, 95%CI: 0.64–2.64, *P* = 0.4763) were not highly correlated to the death of cancer patients with COVID-19 (Additional file 7: Appendix 7).

**Fig. 5:**
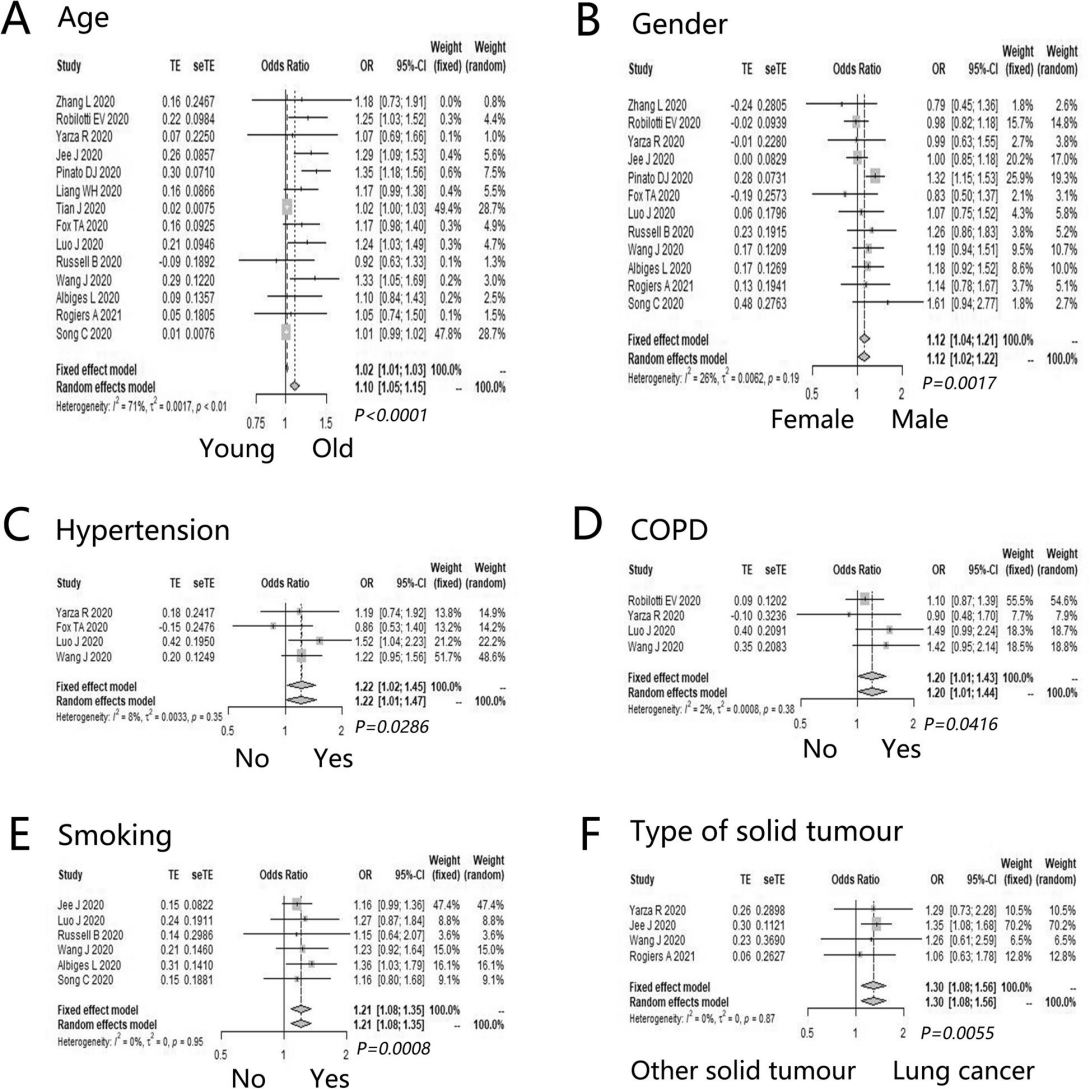
The supernumerary prognostic factors for severe disease of cancer patients with COVID-19. **A** Age (old vs. young); **B** Gender (male vs. female); **C** Hypertension (yes vs. no); **D** COPD (yes vs. no); **E** Smoking (yes vs. no); **F** Type of solid tumor (lung cancer vs. other solid tumor)

Furthermore, the potential prognostic factors for severe disease of cancer patients with COVID-19 included age (OR = 1.10, 95%CI: 1.05–1.15, *P* < 0.0001) (Fig. 5A), gender (OR = 1.12, 95%CI: 1.04–1.21, *P* = 0.0017) (Fig. 5B), hypertension (OR = 1.22, 95%CI: 1.02–1.45, *P* = 0.0286) (Fig. 5C), COPD (OR = 1.20, 95%CI: 1.01–1.43, *P* = 0.0416) (Fig. 5D), smoking (OR = 1.21, 95%CI: 1.08–1.35, *P* = 0.0008) (Fig. 5E), and lung cancer (OR = 1.30, 95%CI: 1.08–1.56, *P* = 0.0055) (Fig. 5F). However, such factors as diabetes (OR = 1.03, 95%CI: 0.88–1.20, *P* = 0.7415), obesity status (OR = 1.00, 95%CI: 0.92–1.10, *P* = 0.9254), ECOG PS (OR = 1.39, 95%CI: 0.93–2.07, *P* = 0.1119), white blood cell count (OR = 1.90, 95%CI: 0.88–4.11, *P* = 0.1026), CRP (OR = 1.39, 95%CI: 0.77–2.50, *P* = 0.2735), lymphocyte count (OR = 1.02, 95%CI: 0.76–1.36, *P* = 0.9093), D-dimer (OR = 1.05, 95%CI: 0.98–1.13, *P* = 0.1387), and creatine kinase (OR = 1.52, 95%CI: 0.83–2.77, *P* = 0.1762) did not obviously influence the severe disease of cancer patients with COVID-19 (Additional file 7: Appendix 7).

#### Subgroup analysis

Depending on the type of cancer, patients were further assigned into groups of solid tumor and haematological malignancy, as listed in Additional file 8: Appendix 8.

The potential prognostic factors for the death of solid tumor patients with COVID-19 included age (OR = 1.01, 95%CI: 1.00–1.01, *P* = 0.0168), gender (OR = 1.22, 95%CI: 1.09–1.36, *P* = 0.0006), hypertension (OR = 1.20, 95%CI: 1.00–1.42, *P* = 0.0446), and smoking (OR = 1.19, 95%CI: 1.04–1.35, *P* = 0.0110).

Furthermore, age (OR = 1.37, 95%CI: 1.20–1.57, *P* < 0.0001), hypertension (OR = 1.20, 95%CI: 1.02–1.41, *P* = 0.0246) and diabetes (OR = 1.26, 95%CI: 1.03–1.53, *P* = 0.0245) ranked as the potential prognostic factors for the death of haematological malignancy patients with COVID-19.

## Discussion

A meta-analysis involving 15 studies demonstrated that chemotherapy could increase the risk of death from COVID-19 in cancer patients [61]. To our best knowledge, this study composed of 52 cohorts involving 9231 cancer patients with COVID-19, was so far the largest-scale investigation with respect to the impact of anti-tumor approaches on clinical outcomes of cancer patients with COVID-19, indicating that cancer patients with recent anti-tumor therapy (especially chemotherapy) were generally susceptible to develop into severe COVID-19, or even death.

Firstly, cancer patients with COVID-19 receiving chemotherapy were more likely to confront with severe disease and death, probably because patients treated with chemotherapy were susceptible to suffer from bone marrow suppression (including severe neutropenia or lymphocytopenia) and impaired immunity [62, 63], even respiratory infections (involving viral etiology) [64]. Furthermore, the recovery of immune system might take a long time after the weakening of immune functions by chemotherapy [65]. As a result, cancer patients with COVID-19 failed to effectively activate the immune system to eliminate the virus in a timely manner [66], that’s why they were more likely to trigger severe disease or even death.

Secondly, recent surgery might lead to increasing risk of death and a trend of severe disease in cancer patients with COVID-19, partially attributable to their frequent visits to hospital and postoperative negative nitrogen balance. Moreover, the stress and trauma caused by surgery could be clinically manifested as decreased immunity, since numerous studies revealed that the immunity of patients would reduce to a certain extent in a period of time after surgery [67].

Thirdly, patients administered with targeted therapy before COVID-19 diagnosis faced with elevated risk of severe disease. Despite targeted therapy seldomly impaired the immunity system of cancer patients, all those receiving maintenance targeted therapy suffered from advanced disease and many complications in general, giving rise to clinical worsening as a result.

Finally, tumor immunotherapy has played an increasingly crucial role in the field of anti-tumor treatment over the past decade [68]. As shown in our study, cancer patients with COVID-19 who received immunotherapy recently did not generate a higher rate of severe disease or death when comparing to those without immunotherapy.

In summary, this study aimed at providing clinicians with preliminary evidence for the safety of anti-tumor approaches during COVID-19. As to patients with COVID-19 who received anti-tumor approaches recently, especially chemotherapy, surgery and targeted therapy, clinicians should focus on disease progression and make intervention in a timely manner when necessary. Furthermore, intensive nursing and positive measures shall be taken to improve the prognosis and reduce the risk of death in practice.

### Limitations

This study came up with four drawbacks as follows: firstly, limited studies related to radiotherapy, surgery and endocrine therapy might affect the accuracy of pooled results to some degree; secondly, 23 included studies failed to separate solid tumor from haematological malignancy for investigating the impact of anti-tumor approaches on the clinical outcomes, which might influence the accuracy of results; thirdly, bias might exist to some extent for excluding relevant studies published in non-English language; lastly, other forms of bias should be taken into account as follows: position bias (e.g. different health care systems and national policies in managing COVID-19) and time lag bias (time of study: start of pandemic vs. later phase of pandemic), which were not available in the included studies.

## Conclusions

Anti-tumor therapy, especially chemotherapy, augmented the risk of severe disease and death for cancer patients with COVID-19, so did surgery for the risk of death and targeted therapy for the incidence of severe COVID-19.

## Supplementary Information

**Additional file 1.**

**Additional file 2.**

**Additional file 3.**

**Additional file 4.**

**Additional file 5.**

**Additional file 6.**

**Additional file 7.**

**Additional file 8.**

## Data Availability

The datasets generated during and/or analyzed during the current study are available from the corresponding author upon reasonable request.

## Abbreviations

COVID-19: Coronavirus disease 2019
ORs: Odds ratios
CIs: Confidence intervals
COPD: Chronic obstructive pulmonary disease
SARS-CoV-2: Severe acute respiratory syndrome-related coronavirus 2
NOS: Newcastle-Ottawa Scale
ECOG PS: Eastern Cooperative Oncology Group Performance Scale
NLR: Neutrophil to lymphocyte ratio
ICIs: Immune checkpoint inhibitors
T-PCR: Reverse transcription-polymerase chain reaction
NA: Not available
ICU: Intensive care unit
