# Supplementary material for: The impact of anti-tumor approaches on the outcomes of cancer patients with COVID-19: a meta-analysis based on 52 cohorts incorporating 9231 participants
Source: BMC Cancer. 2022 Mar 4;22:241. doi: 10.1186/s12885-022-09320-x (PMC8895689; doi:10.1186/s12885-022-09320-x)
Supplement: Supplementary file 1 — Additional file 1. [file 12885_2022_9320_MOESM1_ESM.docx]

**Appendix 1 The full search strategy**

**(((((**COVID-19**) OR (**SARS-CoV2**)) OR (**SARS-CoV-2**)) OR (**2019-nCoV**)) OR (**novel coronavirus**)) AND (((((**cancer**) OR (**neoplasm**)) OR (**malignancy**)) OR (**carcinoma**)) OR (**tumor**))**
