# Supplementary material for: The impact of anti-tumor approaches on the outcomes of cancer patients with COVID-19: a meta-analysis based on 52 cohorts incorporating 9231 participants
Source: BMC Cancer. 2022 Mar 4;22:241. doi: 10.1186/s12885-022-09320-x (PMC8895689; doi:10.1186/s12885-022-09320-x)
Supplement: Supplementary file 3 — Additional file 3. [file 12885_2022_9320_MOESM3_ESM.docx]

**Appendix 3 The Newcastle-Ottawa scale for quality assessment of the studies.**

| Included study | Selection | | | | Comparability | Exposure/outcome | | | Total scores |
| --- | --- | --- | --- | --- | --- | --- | --- | --- | --- |
|  | Is the case definition adequate?/ ascertainment of exposure | Representativeness of the cases/ exposed cohort | Selection of controls/the non-exposed cohort | Definition of controls/demonstration that outcome of interest was not present at start of study | Comparability of both groups/ cohorts on the basis of the design or analysis | Ascertainment of exposure/ assessment of outcome | same method of ascertainment for both groups/ was follow-up long enough for outcomes to occur | Non-response rate/ adequacy of follow up of cohorts |  |
| Kuderer NM ^6^ | * | * | * | * | ** | * | * | * | 9 |
| Lee LYW ^19^ | * | * | * | * | ** | * | * | * | 9 |
| Zhang L ^14^ | * | * | * | - | * | * | * | - | 6 |
| Stroppa EM ^20^ | * | * | * | - | * | * | * | * | 7 |
| Yang K ^7^ | * | * | * | - | * | * | * | * | 7 |
| Zhang H ^21^ | * | * | * | - | * | * | * | * | 7 |
| Robilotti EV ^22^ | * | * | * | - | * | * | * | * | 7 |
| Yarza R ^23^ | * | * | - | * | ** | * | * | * | 8 |
| Li Q ^24^ | * | * | * | - | * | * | * | - | 6 |
| Jee J ^25^ | * | * | * | - | * | * | * | * | 7 |
| Sanchez-Pina JM ^26^ | * | * | * | - | * | * | * | - | 6 |
| Pinato DJ ^15^ | * | * | * | - | * | * | * | * | 7 |
| Assaad S ^27^ | * | * | * | - | * | * | * | * | 7 |
| Garassino MC ^28^ | * | * | * | - | * | * | * | * | 7 |
| Liang WH ^29^ | * | * | * | - | * | * | * | * | 7 |
| Ma J ^30^ | * | * | * | - | * | * | * | * | 7 |
| Mehta V ^11^ | * | * | * | - | * | * | * | - | 6 |
| Yu J ^31^ | * | * | * | - | * | * | * | - | 6 |
| Tian J ^4^ | * | * | - | - | * | * | * | * | 6 |
| Fox TA ^32^ | * | * | * | - | * | * | * | * | 7 |
| Booth S ^33^ | * | * | * | * | ** | * | * | * | 9 |
| Cattaneo C ^34^ | * | * | * | - | * | * | * | - | 6 |
| Lara OD ^35^ | * | * | * | - | * | * | * | - | 6 |
| Liu C ^36^ | * | * | * | - | * | * | * | * | 7 |
| Luo J ^37^ | * | * | * | - | * | * | * | * | 7 |
| Mato AR ^38^ | * | * | * | - | * | * | * | * | 7 |
| Rogado J ^39^ | * | * | * | - | * | * | * | * | 7 |
| Russell B ^40^ | * | * | * | * | ** | * | * | * | 9 |
| Scarfò L ^41^ | * | * | * | - | * | * | * | * | 7 |
| Vuagnat P ^42^ | * | * | * | - | * | * | * | * | 7 |
| Wang BO ^43^ | * | * | * | - | * | * | * | * | 7 |
| Wang J ^44^ | * | * | * | - | * | * | * | * | 7 |
| Gonzalez-cao M ^45^ | * | * | * | - | * | * | * | - | 6 |
| De Melo AC ^46^ | * | * | * | - | * | * | * | * | 7 |
| Albiges L ^47^ | * | * | * | - | * | * | * | * | 7 |
| Martínez-López J ^48^ | * | * | * | - | * | * | * | - | 6 |
| Martín-Moro F ^49^ | * | * | * | - | * | * | * | * | 7 |
| Lattenist R ^50^ | * | * | * | - | * | * | * | - | 6 |
| Nakamura S ^51^ | * | * | * | - | * | * | * | - | 6 |
| Rogiers A ^52^ | * | * | * | - | * | * | * | * | 7 |
| Glenthøj A ^16^ | * | * | * | * | ** | * | * | * | 9 |
| Song C ^17^ | * | * | - | - | * | * | * | - | 5 |
| Lunski MJ ^18^ | * | * | * | - | * | * | * | - | 6 |
| Nie L ^53^ | * | * | * | - | * | * | * | - | 6 |
| Larfors G ^54^ | * | * | * | - | * | * | * | - | 6 |
| H€ollein A ^55^ | * | * | * | - | * | * | * | - | 6 |
| Garnett C ^56^ | * | * | * | - | * | * | * | * | 7 |
| Hanna GJ ^57^ | * | * | * | - | * | * | * | * | 7 |
| Lie`vre A ^58^ | * | * | * | * | ** | * | * | * | 9 |
| Smith M ^59^ | * | * | * | - | * | * | * | - | 6 |
| Wu YG ^60^ | * | * | * | - | * | * | * | * | 7 |
| Yang F ^12^ | * | * | * | - | * | * | * | - | 6 |
