# Supplementary material for: The impact of anti-tumor approaches on the outcomes of cancer patients with COVID-19: a meta-analysis based on 52 cohorts incorporating 9231 participants
Source: BMC Cancer. 2022 Mar 4;22:241. doi: 10.1186/s12885-022-09320-x (PMC8895689; doi:10.1186/s12885-022-09320-x)
Supplement: Supplementary file 4 — Additional file 4. [file 12885_2022_9320_MOESM4_ESM.docx]

**Appendix 4 Publication bias for Anti-tumor therapy (Egger’s test)**

| **Anti-tumor therapy** | **Death** | | |  | **Severe COVID-19** | | |
| --- | --- | --- | --- | --- | --- | --- | --- |
|  | **Total** | **Solid tumour** | **Haematological malignancy** |  | **Total** | **Solid tumour** | **Haematological malignancy** |
| Chemotherapy | 0.3820 | 0.6242 | 0.4969 |  | 0.5418 | 0.4969 | NA |
| Radiotherapy | 0.1742 | NA | NA |  | NA | NA | NA |
| Targeted therapy | 0.8046 | NA | NA |  | 0.5730 | NA | NA |
| Surgery | 0.4969 | NA | NA |  | 0.1172 | NA | NA |
| Endocrine therapy | 0.1172 | NA | NA |  | NA | NA | NA |
| Immunotherapy | 0.5312 | 0.4969 | NA |  | 0.4579 | NA | NA |
| Anti-tumor therapy | 0.5516 | 0.8806 | 0.3223 |  | 0.3930 | 0.2931 | NA |
