# Supplementary material for: The impact of anti-tumor approaches on the outcomes of cancer patients with COVID-19: a meta-analysis based on 52 cohorts incorporating 9231 participants
Source: BMC Cancer. 2022 Mar 4;22:241. doi: 10.1186/s12885-022-09320-x (PMC8895689; doi:10.1186/s12885-022-09320-x)
Supplement: Supplementary file 5 — Additional file 5. [file 12885_2022_9320_MOESM5_ESM.docx]

**Appendix 5 Publication bias for supernumerary prognostic factors (Egger’s test)**

| **Prognostic factors** | **Death** | | |  | **Severe COVID-19** | | |
| --- | --- | --- | --- | --- | --- | --- | --- |
|  | **Total** | **Solid tumour** | **Haematological malignancy** |  | **Total** | **Solid tumour** | **Haematological malignancy** |
| Age (old vs. young) | 0.7745 | 0.6207 | 0.1885 |  | 0.4115 | 1.0000 | NA |
| Gender (male vs. female) | 0.9368 | 0.2931 | 1.0000 |  | 0.6808 | 0.1742 | NA |
| Hypertension (yes vs. no) | 0.4641 | 0.1742 | 0.3272 |  | 0.1742 | NA | NA |
| Diabetes (yes vs. no) | 0.2429 | 0.6015 | 0.6015 |  | 0.5730 | NA | NA |
| COPD (yes vs. no) | 0.8806 | 1.0000 | NA |  | 0.4969 | NA | NA |
| Cardiovascular disease (yes vs. no) | 1.0000 | NA | NA |  | NA | NA | NA |
| Obesity status (yes vs. no) | 0.8510 | NA | NA |  | 0.5730 | NA | NA |
| Smoking (yes vs. no) | 0.3520 | 0.6523 | NA |  | 0.8510 | NA | NA |
| ECOG PS (high vs. low) | 0.0953 | 0.1742 | NA |  | 1.0000 | NA | NA |
| Type of solid tumour (lung cancer vs. other solid tumour) | 0.5730 | 0.6015 | NA |  | 0.4969 | NA | NA |
| White blood cell count (high vs. normal) | 1.0000 | NA | NA |  | 0.6015 | NA | NA |
| C-reactive protein (high vs. normal) | 0.5316 | NA | NA |  | 0.6015 | NA | NA |
| Lymphocyte count (high vs. normal) | 0.2931 | NA | NA |  | 0.6242 | NA | NA |
| D-dimer (high vs. normal) | 0.4969 | NA | NA |  | 0.1172 | NA | NA |
| NLR (high vs. normal) | 0.6015 | NA | NA |  | NA | NA | NA |
| Creatine kinase (high vs. normal) | NA | NA | NA |  | 0.6015 | NA | NA |

Abbreviations: COPD, chronic obstructive pulmonary disease; ECOG PS, Eastern Cooperative Oncology Group Performance Scale; NLR, neutrophil to lymphocyte ratio; NA, not available.
