# Supplementary material for: The impact of anti-tumor approaches on the outcomes of cancer patients with COVID-19: a meta-analysis based on 52 cohorts incorporating 9231 participants
Source: BMC Cancer. 2022 Mar 4;22:241. doi: 10.1186/s12885-022-09320-x (PMC8895689; doi:10.1186/s12885-022-09320-x)
Supplement: Supplementary file 6 — Additional file 6. [file 12885_2022_9320_MOESM6_ESM.docx]

**Appendix 6 The impact of anti-tumor therapy on death and severe disease of cancer patients with COVID-19**


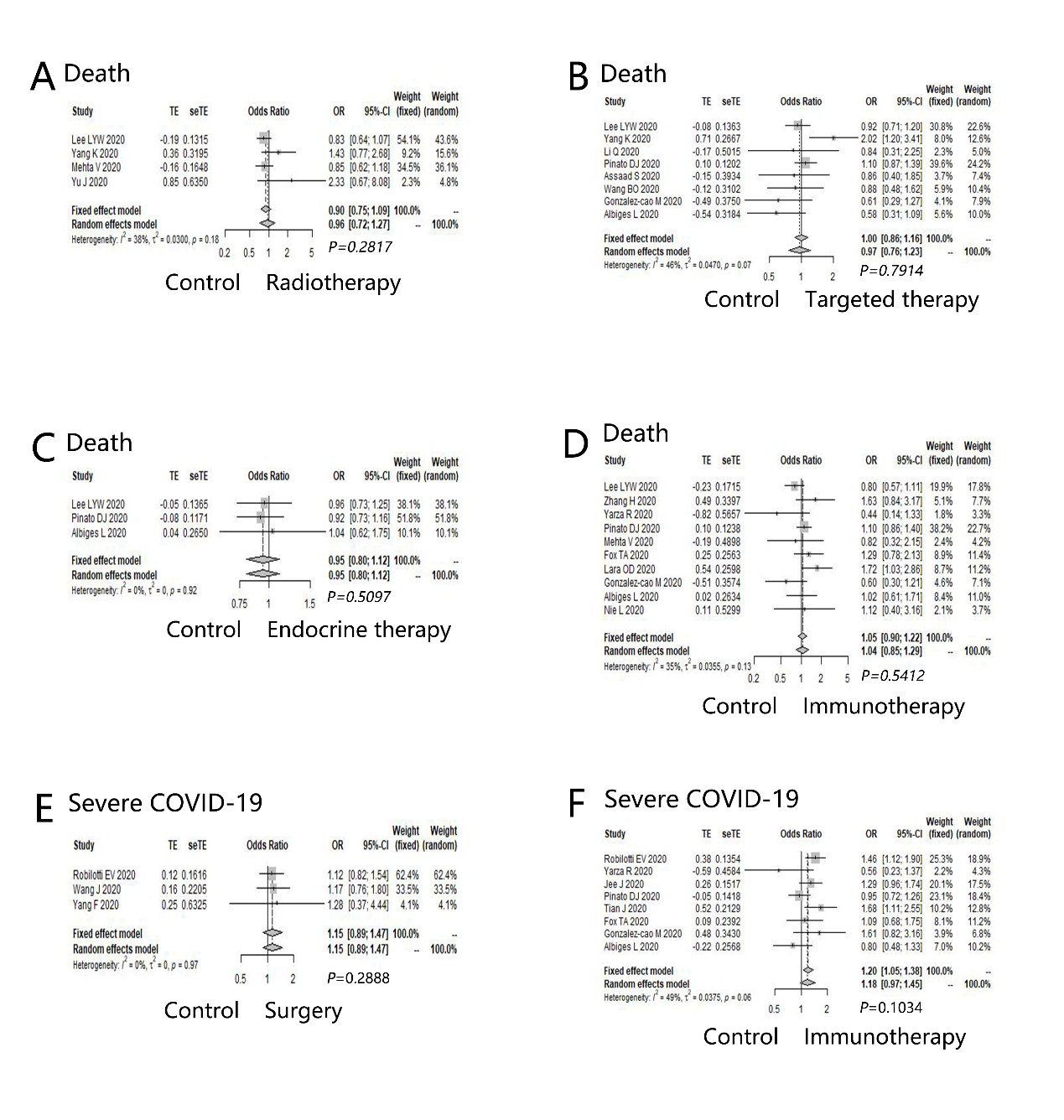


The impact of (A) radiotherapy, (B) targeted therapy, (C) endocrine therapy, and (D) immunotherapy on death of cancer patients with COVID-19; The impact of (E) surgery and (F) immunotherapy on severe disease of cancer patients with COVID-19.
