# Supplementary material for: The impact of anti-tumor approaches on the outcomes of cancer patients with COVID-19: a meta-analysis based on 52 cohorts incorporating 9231 participants
Source: BMC Cancer. 2022 Mar 4;22:241. doi: 10.1186/s12885-022-09320-x (PMC8895689; doi:10.1186/s12885-022-09320-x)
Supplement: Supplementary file 7 — Additional file 7. [file 12885_2022_9320_MOESM7_ESM.docx]

**Appendix 7 Supernumerary prognostic factors for death and severe disease of cancer patients with COVID-19**


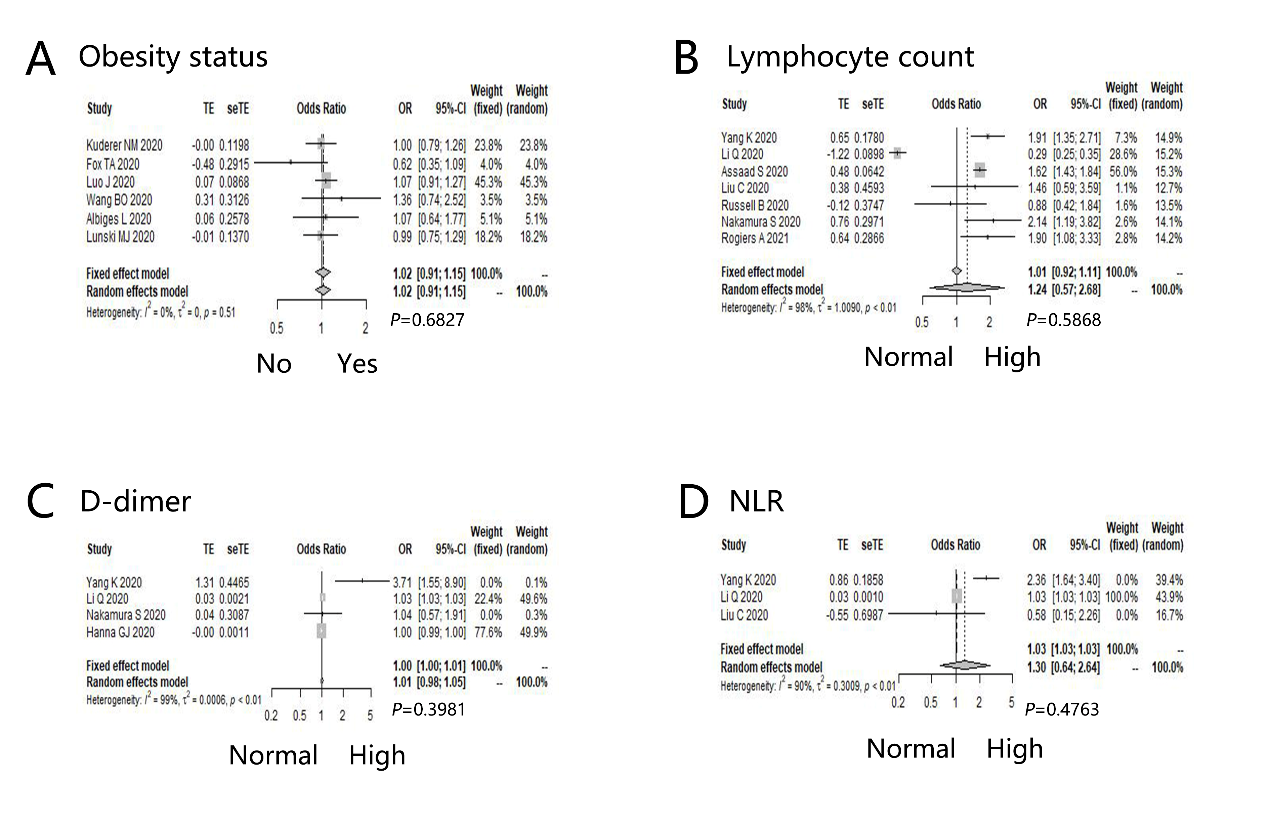


(A) Obesity status (yes vs. no), (B) Lymphocyte count (high vs. normal), (C) D-dimer (high vs. normal), and (D) NLR (high vs. normal) for death of cancer patients with COVID-19.


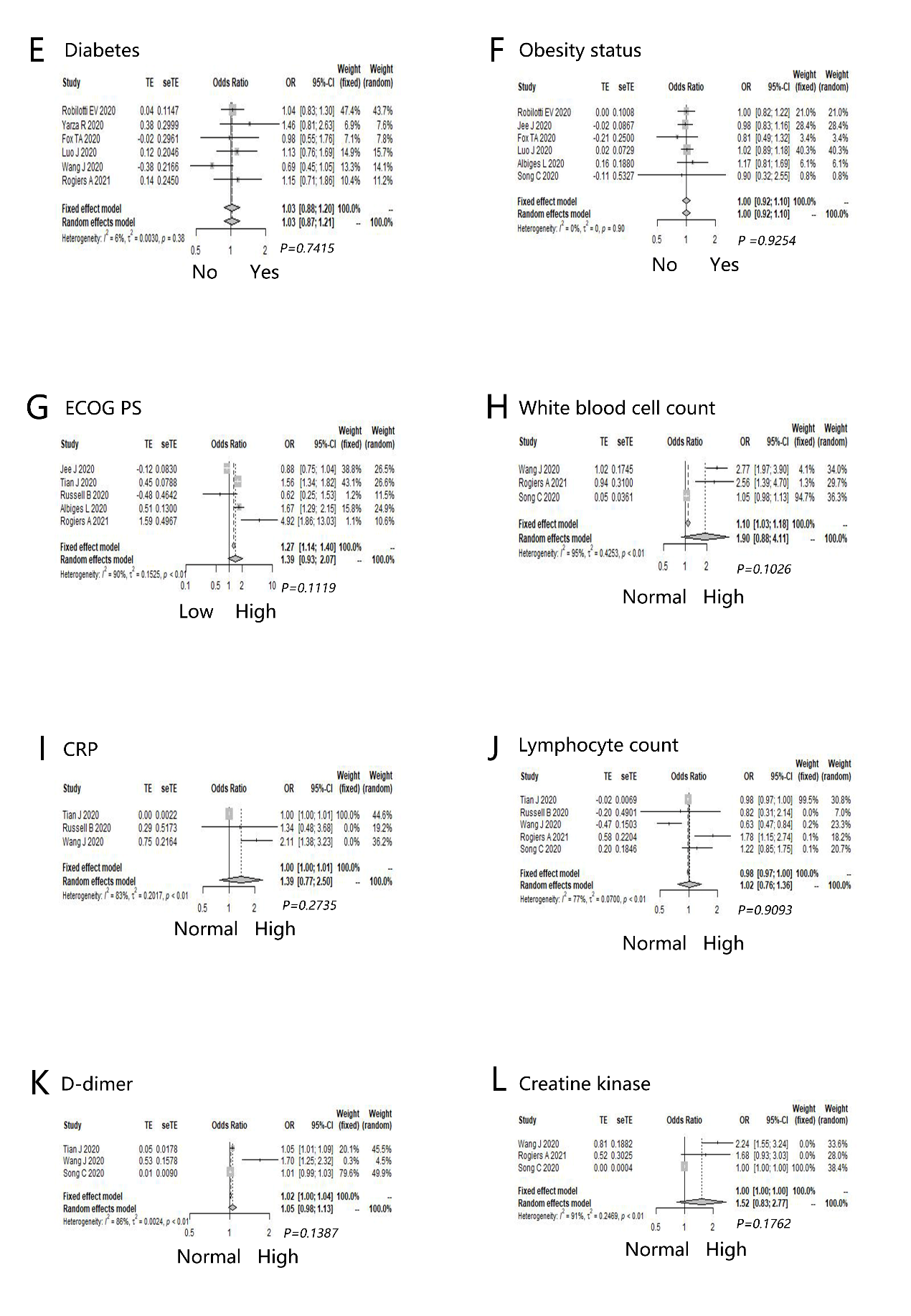


(E) Diabetes (yes vs. no), (F) Obesity status (yes vs. no), (G) ECOG PS (high vs. low), (H) White blood cell count (high vs. normal), (I) C-reactive protein (high vs. normal), (J) Lymphocyte count (high vs. normal), (K) D-dimer (high vs. normal), and (L) Creatine kinase (high vs. normal) for severe disease of cancer patients with COVID-19.
