# Supplementary material for: The impact of anti-tumor approaches on the outcomes of cancer patients with COVID-19: a meta-analysis based on 52 cohorts incorporating 9231 participants
Source: BMC Cancer. 2022 Mar 4;22:241. doi: 10.1186/s12885-022-09320-x (PMC8895689; doi:10.1186/s12885-022-09320-x)
Supplement: Supplementary file 8 — Additional file 8. [file 12885_2022_9320_MOESM8_ESM.docx]

**Appendix 8 Supernumerary prognostic factors for death and severe disease of COVID-19 infected solid tumour and haematological malignancy**

| **Prognostic factors** | **Solid tumour** | | | | **Haematological malignancy** | | | |
| --- | --- | --- | --- | --- | --- | --- | --- | --- |
|  | **death** | | **severe COVID-19** | | **death** | | **severe COVID-19** | |
|  | **OR (95%CI)** | ***P*** | **OR (95%CI)** | ***P*** | **OR (95%CI)** | ***P*** | **OR (95%CI)** | ***P*** |
| Age (old vs. young) | 1.01 (1.00-1.01) | 0.0168 | 1.15 (1.00-1.34) | 0.0562 | 1.37 (1.20-1.57) | <0.0001 | NA | NA |
| Gender (male vs. female) | 1.22 (1.09-1.36) | 0.0006 | 1.06 (0.86-1.30) | 0.6002 | 1.19 (0.84-1.68) | 0.3235 | NA | NA |
| Hypertension (yes vs. no) | 1.20 (1.00-1.42) | 0.0446 | NA | NA | 1.20 (1.02-1.41) | 0.0246 | NA | NA |
| Diabetes (yes vs. no) | 1.07 (0.82-1.40) | 0.6354 | NA | NA | 1.26 (1.03-1.53) | 0.0245 | NA | NA |
| COPD (yes vs. no) | 1.30 (0.90-1.90) | 0.1650 | NA | NA | NA | NA | NA | NA |
| Cardiovascular disease (yes vs. no) | NA | NA | NA | NA | NA | NA | NA | NA |
| Obesity status (yes vs. no) | NA | NA | NA | NA | NA | NA | NA | NA |
| Smoke (yes vs. no) | 1.19 (1.04-1.35) | 0.0110 | NA | NA | NA | NA | NA | NA |
| ECOG PS (high vs. low) | 1.24 (0.84-1.83) | 0.2703 | NA | NA | NA | NA | NA | NA |
| Type of solid tumour (lung cancer vs. other solid tumour) | 1.53 (0.85-2.76) | 0.1555 | NA | NA | NA | NA | NA | NA |
| White blood cell count (high vs. normal) | NA | NA | NA | NA | NA | NA | NA | NA |
| C-reactive protein (high vs. normal) | NA | NA | NA | NA | NA | NA | NA | NA |
| Lymphocyte count (high vs. normal) | NA | NA | NA | NA | NA | NA | NA | NA |
| D-dimer (high vs. normal) | NA | NA | NA | NA | NA | NA | NA | NA |
| NLR (high vs. normal) | NA | NA | NA | NA | NA | NA | NA | NA |
| Creatine kinase (high vs. normal) | NA | NA | NA | NA | NA | NA | NA | NA |

Abbreviations: COPD, chronic obstructive pulmonary disease; ECOG, Eastern Cooperative Oncology Group Performance Scale; NLR, neutrophil to lymphocyte ratio; NA, not available; OR, odds ratio; CI, confidence interval.
